# Cystatin C is not a good candidate biomarker for HNF1A-MODY

**DOI:** 10.1007/s00592-012-0378-1

**Published:** 2012-02-19

**Authors:** Natalia Nowak, Magdalena Szopa, Gaya Thanabalasingham, Tim J. McDonald, Kevin Colclough, Jan Skupien, Timothy J. James, Beata Kiec-Wilk, Elzbieta Kozek, Wojciech Mlynarski, Andrew T. Hattersley, Katharine R. Owen, Maciej T. Malecki

**Affiliations:** 1 Department of Metabolic Diseases, Jagiellonian University Medical College, 15 Kopernika Street, 31-501 Krakow, Poland; 2 Department of Medical Education, Jagiellonian University Medical College, Krakow, Poland; 3 University Hospital, Krakow, Poland; 4 Oxford Centre for Diabetes, Endocrinology and Metabolism, University of Oxford, Churchill Hospital, Oxford, UK; 5 The Oxford NIHR Biomedical Research Centre, Churchill Hospital, Oxford, UK; 6 NIHR Clinical Research Facility, Peninsula College of Medicine and Dentistry, University of Exeter, Exeter, Devon UK; 7 Section on Genetics and Epidemiology, Joslin Diabetes Center, Boston, MA USA; 8 Department of Clinical Biochemistry, John Radcliffe Hospital, Oxford, UK; 9 Department of Paediatrics, Oncology, Hematology and Diabetology, Medical University of Lodz, Lodz, Poland

**Keywords:** Monogenic diabetes, MODY, Cystatin C, HNF1A

## Abstract

Cystatin C is a marker of glomerular filtration rate (GFR). Its level is influenced, among the others, by CRP whose concentration is decreased in HNF1A-MODY. We hypothesized that cystatin C level might be altered in HNF1A-MODY. We aimed to evaluate cystatin C in HNF1A-MODY both as a diagnostic marker and as a method of assessing GFR. We initially examined 51 HNF1A-MODY patients, 56 subjects with type 1 diabetes (T1DM), 39 with type 2 diabetes (T2DM) and 43 non-diabetic individuals (ND) from Poland. Subjects from two UK centres were used as replication panels: including 215 HNF1A-MODY, 203 T2DM, 39 HNF4A-MODY, 170 GCK-MODY, 17 HNF1B-MODY and 58 T1DM patients. The data were analysed with additive models, adjusting for gender, age, BMI and estimated GFR (creatinine). In the Polish subjects, adjusted cystatin C level in HNF1A-MODY was lower compared with T1DM, T2DM and ND (*p* < 0.05). Additionally, cystatin C-based GFR was higher than that calculated from creatinine level (*p* < 0.0001) in HNF1A-MODY, while the two GFR estimates were similar or cystatin C-based lower in the other groups. In the UK subjects, there were no differences in cystatin C between HNF1A-MODY and the other diabetic subgroups, except HNF1B-MODY. In UK HNF1A-MODY, cystatin C-based GFR estimate was higher than the creatinine-based one (*p* < 0.0001). Concluding, we could not confirm our hypothesis (supported by the Polish results) that cystatin C level is altered by *HNF1A* mutations; thus, it cannot be used as a biomarker for HNF1A-MODY. In HNF1A-MODY, the cystatin C-based GFR estimate is higher than the creatinine-based one.

## Introduction

Cystatin C is a low-molecular-weight protein produced at a constant rate in all nucleated cells; it is freely filtered in the renal glomeruli, reabsorbed and catabolized in the proximal tubules [1]. Cystatin C has been shown to be an excellent marker of glomerular filtration rate (GFR) in healthy individuals and various disease states, including diabetes [1, 2]. Mutations in *HNF1A*, encoding hepatocyte nuclear factor-1alpha, are a common cause of maturity-onset diabetes of the young (MODY) in Europeans [3], but not in Asians [4]. This form of diabetes is characterized by impaired insulin secretion [5]. Making an accurate diagnosis of HNF1A-MODY can improve patients’ treatment [6]. Currently, genetic testing is considered too expensive to be widely used. The solution may lie in the development of non-genetic biomarkers that can be used as a more economical screening test to identify those at highest risk of MODY prior to molecular testing.

The recently published report described lower hsCRP levels in HNF1A-MODY [7]. Interestingly, CRP is one of the factors other than GFR that affect cystatin C levels. Elevated CRP has been consistently associated with increased cystatin C [8–10]. Thus, we hypothesized that the serum cystatin C level may be altered by *HNF1A* mutations. Additional justification for this study has come from the fact that the clinical picture of HNF1A-MODY includes several renal phenotypes, including tubulopathy with generalized aminoaciduria and glycosuria [11–13]. The aims of the current study were (1) to evaluate cystatin C as a diagnostic biomarker for HNF1A-MODY and (2) to assess whether cystatin C provides a reliable estimate of GFR in subjects with HNF1A-MODY.

## Methods

In an initial study, we included 51 HNF1A-MODY patients, 56 subjects with type 1 diabetes mellitus (T1DM), 41 with type 2 diabetes mellitus (T2DM) and 43 non-diabetic individuals (ND) from Poland. Subjects from two UK centres were used as replication panels. The Oxford subjects consisted of 36 HNF1A-MODY cases and 36 young-onset T2DM (diagnosed ≤45 years of age) matched for gender, current age and serum creatinine. The Exeter subjects consisted of 179 HNF1A-MODY, 167 T2DM, 39 HNF4A-MODY, 170 GCK-MODY, 17 HNF1B-MODY and 58 T1DM patients. In the UK (Oxford) group, one HNF1A-MODY subject and 5 T2DM subjects were of non-European origin. All the other individuals were European Caucasians.

In all three centres, serum creatinine was analysed using a Jaffe method with calibration aligned to IDMS. In Poland and Exeter, the measurements were done using the Roche P800 analyser (Roche Diagnostics, Burgess Hill, UK), while in Oxford, the ADVIA 2400 device (Siemens Diagnostics, Frimley, UK) was used. Polish samples were analysed for cystatin C using a particle-enhanced assay nephelometric immunoassay (PENIA) with N-Latex cystatin C kit on a BNNII analyser (Dade-Behring, Marburg, Germany). The UK samples were analysed using particle-enhanced turbidimetric immunoassay (PETIA) using either the ADVIA 2400 analyser (Oxford) or the P800 modular system (Exeter). HsCRP was analysed on the Polish samples using immunoturbidimetry.

We estimated GFR from creatinine with the CKD-EPI formula [14] and from cystatin C with the 4-variable formula [1]. The data were analysed with linear regression models, with the addition of estimated nonlinear nonparametric additive components in order to relax the linearity assumption, when adjusting for confounders. *p* values <0.05 were considered significant. The statistical analysis was performed with SAS 9.2.

## Results

The study groups’ characteristics are shown in Table 1. In the Polish cohort, we identified 3 HNF1A-MODY patients with chronic kidney disease (CKD) defined as CKD-EPI estimated GFR (eGFR) <60 ml/min/1.73 m^2^ [15]; there were 5 such individuals in the T1DM group, 6 in the T2DM group and none in the ND group. Cystatin C level in HNF1A-MODY was similar to that observed in ND individuals (*p* = 0.95) and significantly lower than in the other diabetic groups (*p* < 0.0001 for both groups). Adjusting for differences in CKD-EPI eGFR, gender, age and BMI revealed a significantly lower cystatin C level in HNF1A-MODY compared with T1DM, T2DM and ND groups (0.197 mg/l less than T1DM, 95% CI: 0.138–0.257, *p* < 0.0001; 0.148 mg/l less than T2DM, 95% CI: 0.083–0.214, *p* < 0.0001; and 0.069 mg/l less than ND, 95% CI: 0.06–0.133, *p* < 0.03). With adjustment for eGFR only and with adjustment to HbA1c among diabetic groups, the pattern of mean cystatin C levels and statistical significance of differences remained unchanged.

**Table 1:** Characteristics of the study groups

	HNF1A-MODY	T1DM	T2DM	GCK	HNF4A	HNF1B	ND
*Polish subjects*							
*N*	52	56	41		–	–	43
% Female	57.7	66.1	61.0	–	–	–	60.5
Age [years]	36.5 (32.2–40.9)	36.1 (33.1–39.2)	57.9 (55.1–60.7)	–	–	–	32.3 (28.4–36.3)
Diabetes duration [years]	11.5 (8.3–14.7)	16.4 (13.8–18.9)	10.1 (7.4–12.7)	–	–	–	–
BMI [kg/m^2^]	22.5 (21.7–23.4)	23.6 (22.6–24.7)	33.9 (31.5–36.3)	–	–	–	23.6 (22.2–24.9)
HbA1c mmol/mol [%]	54 (50–58) 7.1 (6.7–7.5)	58 (54–63) 7.5 (7.1–7.9)	70 (63–78) 8.6 (7.9–9.3)	–	–	–	–
Creatinine [μmol/l] Range	74.8 (69.6–80.1) 47.1–146.3	75.3 (69.2–81.4) 50.2–166.0	79.3 (71.9–86.6) 47.9–160.2	–	–	–	67.3 (63.4–71.1) 46.1–93.5
Cystatin C [mg/l] Range	0.74 (0.69–0.79) 0.47–1.67	0.93 (0.86–0.99) 0.51–1.73	0.99 (0.89–1.09) 0.60–2.29	–	–	–	0.74 (0.70–0.78) 0.52–1.07
CRP [mg/l]^‡^	0.084 (0.033–0.136)	2.041 (1.180–2.903)	0.931 (0.316–1.546)	–	–	–	0.263 (0.074–0.452)
EGFR CKD–EPI [ml/min/1.73 m^2^] Range	102.1 (95.5–108.7) 47.2–161.9	101.2 (95.2–107.2) 30.1–131–7	82.4 (75.3–89.5) 28.1–118.3	–	–	–	111.9 (107.2–116.7) 72.6–155.6
eGFR cysC [ml/min/1.73 m^2^] Range	115.9 (108.7–123.1) 42.0–177.1	88.7 (82.4–95.0) 37.0–169.0	79.5 (71.7–87.4) 25.4–130.8	–	–	–	113.3 (107.5–119.1) 72.6–155.6
*Oxford subjects*				–			
*N*	36	–	36		–	–	–
% Female	66.7	–	66.7	–	–	–	–
Age [years]	42.5 (37.1–47.9)	–	46.1 (42.2–50.0)	–	–	–	–
Diabetes duration [years]	17.4 (11.9–22.9)		10.8 (8.1–13.5)	–	–	–	
BMI [kg/m^2^]	25.7 (23.7–27.6)	–	30.8 (28.6–33.0)	–	–	–	–
Creatinine [μmol/l] Range	86.6 (80.6–92.5) 52.0–136.0	–	86.1 (80.8–91.3) 63.6–135.3	–	–	–	–
Cystatin C [mg/l] Rrange	0.78 (0.69–0.86) 0.51–1.72	–	0.77 (0.71–0.83) 0.49–1.34	–	–	–	–
eGFR CKD-EPI [ml/min/1.73 m^2^] Range	82.1 (75.5–88.8) 36.3–124.1	–	81.3 (75.6–86.9) 42.7–110.5	–	–	–	–
eGFR cysC [ml/min/1.73 m^2^] Range	109.3 (98.8–120.2) 36.8–169.4	–	107.8 (97.4–116.7) 50.2–161.0	–	–	–	–
*Exeter subjects*				–			
*N*	179	58	167	170	39	17	–
% Female	58.7	51.7	43.7	67.1	74.4	64.7	–
Age [years]	38.4 (35.9–40.8)	42.9 (38.5–47.2)	71.0 (69.4–72.6)	34.9 (32.1–37.8)	33.6 (27.5–39.6)	27.1 (18.3–35.8)	–
Diabetes duration [years]	16.6 (14.7–18.5)	21.6 (16.7–26.4)	9.5 (8.3–10.6)	9.5 (7.6–11.3)	12.1 (8.1–16.0)	10.3 (3.0–17.6)	–
BMI [kg/m^2^]	25.1 (24.1–26.2)	24.9 (23.9–25.8)	28.6 (27.7–29.4)	23.1 (22.3–23.9)	24.6 (22.2–27.1)	25.7 (18.4–32.9)	–
Creatinine [μmol/l] Range	85.4 (76.8–93.9) 32.0–730.0	75.2 (71.9–78.4) 44.0–109.0	88.3 (84.4–92.2) 33.0–249.0	77.1 (74.3–79.9) 34.0–142.0	76.0 (68.5–83.4) 22.0–148.0	132.5 (68.1–196.9) 33.0–561.0	–
Cystatin C [mg/l] Range	0.90 (0.82–0.99) 0.40–7.33	0.78 (0.74–0.82) 0.40–1.26	1.1 (1.02–1.15) 0.47–3.63	0.85 (0.82–0.88) 0.40–2.03	0.89 (0.80–0.99) 0.51–1.84	1.55 (1.02–2.07) 0.60–5.19	–
eGFR CKD-EPI [ml/min/1.73 m^2^] Range	93.7 (89.8–97.6) 7.3–181.2	96.6 (91.6–101.3) 56.1–142.4	71.0 (68.0–73.9) 17.9–144.5	97.9 (93.3–102.4) 37.6–193.5	100.5 (88.9–112.0) 32.2–196.7	86.1 (58.8–113.3) 6.4–169.8	–
eGFR cysC [ml/min/1.73 m^2^] Range	99.1 (94.4–103.7) 7.7–234.9	106.1 (98.9–113.3) 58.4–236.6	71.4 (67.8–74.9) 14.4–186.8	99.1 (95.0–103.2) 30.7–224.3	97.8 (88.0–107.7) 37.5–179.7	65.5 (48.8–82.2) 9.9–133.6	–

Using a linear model, instead of additive as the sensitivity analysis, did not change the conclusions or statistical significance, nor did exclusion of subjects with CKD. To further investigate the observed reduced cystatin C levels in HNF1A-MODY, we compared the patients’ GFR estimated with the CKD-EPI formula with the GFR estimated from cystatin C (Table 1). The crude differences between creatinine and cystatin C-based estimated GFRs were not significant in ND individuals (the CKD-EPI GFR estimate was 1.4 ml/min/1.73 m^2^ less, 95% CI: −7.3–4.6), nor in T2DM (the CKD-EPI estimate was 2.0 ml/min/1.73 m^2^ higher, 95% CI: −4.2–8.3). In T1DM, the CKD-EPI estimate was 12.5 ml/min/1.73 m^2^ higher (95% CI: 7.3–17.7, *p* < 0.0001), while in the HNF1A-MODY group the difference was in the opposite direction: the CKD-EPI estimate was 13.8 ml/min/1.73 m^2^ less than cystatin C-based GFR (95% CI: 8.3–19.3, *p* < 0.0001). These results were not altered by adjusting for possible confounders.

Concordant with recent publications [7], HNF1A-MODY subjects had substantially lower hsCRP levels compared with other diabetes subtypes, but also to non-diabetic individuals (*p* < 0.0001, for all pairwise comparisons). CRP was a significant predictor of cystatin C concentration (*p* < 0.0001). The pattern of differences in creatinine and cystatin C GFR estimates was in line with the pattern of mean serum CRP concentrations measured in the groups. Specifically, in the groups with the highest (T1DM) and the lowest (HNF1A-MODY) CRP values, we observed, respectively, a reduced and an increased cystatin C-based GFR estimate, as compared with creatinine-based CKD-EPI estimates.

We subsequently attempted to replicate these findings in the UK subjects. We observed no differences between cystatin C concentrations in HNF1A-MODY and T2DM patients from Oxford (*p* = 0.8), either before or after adjusting for covariates (as in Polish cohort). In both Oxford subject groups, we observed similar large differences between cystatin C-based and creatinine-based GFR estimates: the cystatin C-based measure was 27.4 ml/min/1.73 m^2^ (95% CI: 19.9–34.9; *p* < 0.0001) higher in HNF1A-MODY and 25.8 ml/min/1.73 m^2^ (95% CI: 18.2–33.3; *p* < 0.0001) higher in T2DM.

In the Exeter subjects, there were no significant differences in cystatin C (adjusting for CKD-EPI eGFR, sex, age and BMI) between HNF1A-MODY and T1DM (*p* = 0.3), T2DM (*p* = 0.1), GCK-MODY (*p* = 0.4) and HNF4A-MODY (*p* = 0.9). The HNF1B-MODY subjects had significantly higher cystatin C, by 0.333 mg/l (95% CI: 0.178–0.488), compared with HNF1A-MODY (*p* < 0.0001). In the HNF1A-MODY group, the cystatin C-based GFR estimate was higher than the creatinine-based one by 5.3 ml/min/1.73 m^2^ (95% CI: 1.6–9.1, *p* = 0.005). Thus, this finding was consistent in all three HNF1A-MODY groups. A higher cystatin C-based GFR estimate was also observed in the T1DM subjects recruited through Exeter; the difference between GFR estimates was 9.6 ml/min/1.73 m^2^ (95% CI: 3.1–16.2, *p* = 0.004). There was no statistically significant difference in the GFR estimates in the HNF4A-MODY (*p* = 0.5), GCK-MODY (*p* = 0.5) and T2DM (*p* = 0.8) groups, while in the HNF1B-MODY subjects, the cystatin C-based GFR estimate was lower (*p* = 0.0009), consistent with the significantly higher cystatin C concentration observed in this subgroup.

## Discussion

Our initial results from the Polish population showed that cystatin C concentration in HNF1A-MODY was reduced by approximately 10% compared with the non-diabetic individuals. This is in contrast to the increased cystatin C level previously reported in common forms of diabetes [8] and observed in the Polish subjects in this study.

The current research was initially driven by the report on the decreased CRP level, an important regular of cystatin C level [7], and evidence of abnormalities of renal tubular function in HNF1A-MODY subjects and a Fanconi’s syndrome in an animal model [11, 12, 16]. We considered that in HNF1A-MODY, lower cystatin C levels in the Polish cohort could be, at least partially, mediated by reduced CRP levels. The reduction in cystatin C in these subjects would translate into overestimated GFR compared with creatinine-based estimates.

In the UK replication study, we did not observe a difference in cystatin C levels between the UK HNF1A-MODY and other diabetes types, except HNF1B-MODY. Those with *HNF1B* mutations have predominantly developmental renal anomalies and renal dysfunction; however, as the results were adjusted for GFR, this does not fully explain the difference. This observation warrants further confirmation. The reason for the heterogeneity between the results from the Polish and UK centres is unclear; one possible explanation is a random variation and false positive result in the initial group. Different cystatin C assays used at the different sites may have contributed [17], but this is more likely to have resulted in between-site bias rather than the observed between-group differences. Differences in recruitment procedures and study group characteristics also cannot be ruled out. Whilst there are known limitations in calculation protocols for estimating GFR [18], the consistent observation in all three HNF1A-MODY groups was that GFR calculated using a cystatin C-based formula was higher than those based on creatinine. This should be taken into account in the clinical management of HNF1A-MODY patients. The important strength of this study is the large HNF1A-MODY sample size and attempt to replicate in independent cohorts. The shortcoming is the fact that MODY subjects were compared to relatively small groups of patients with T1DM and T2DM who differed in terms of some clinical features. On the other hand, the statistical analysis chosen in this paper should have adjusted for these differences, some of which, such as age of onset and BMI, inevitably associated with the clinical picture of examined types of diabetes.

In summary, we were not able to confirm our hypothesis (supported by the results from the Polish subjects) that cystatin C level is altered by *HNF1A* mutations; thus, this molecule cannot be considered a candidate biomarker for HNF1A-MODY. Additionally, in this form of diabetes, the cystatin C-based GFR estimate is higher than the creatinine-based one.
